# Health Impacts and Economic Costs of Air Pollution in the Metropolitan Area of Skopje

**DOI:** 10.3390/ijerph15040626

**Published:** 2018-03-29

**Authors:** Gerardo Sanchez Martinez, Joseph V. Spadaro, Dimitris Chapizanis, Vladimir Kendrovski, Mihail Kochubovski, Pierpaolo Mudu

**Affiliations:** 1 The UNEP-DTU Partnership, Department of Management Engineering, Technical University of Denmark, UN City, Marmorvej 51, 2100 Copenhagen Ø, Denmark; gsama@dtu.dk; 2 WHO European Centre for Environment and Health, Platz der Vereinten Nationen 1, 53113 Bonn, Germany; kendrovskiv@who.int; 3 SERC, Hillsborough, NJ 08844, USA; 4 Environmental Engineering Laboratory, Department of Chemical Engineering, Aristotle University of Thessaloniki, 54124 Thessaloniki, Greece; dimitris@eng.auth.gr; 5 Institute of Public Health, 50 Divizija 6, 1000 Skopje, The former Yugoslav Republic of Macedonia; kocubov58@gmail.com

**Keywords:** air pollution, Skopje, the former Yugoslav Republic of Macedonia, particulate matter, economic evaluation, burden of disease

## Abstract

Background: Urban outdoor air pollution, especially particulate matter, remains a major environmental health problem in Skopje, the capital of the former Yugoslav Republic of Macedonia. Despite the documented high levels of pollution in the city, the published evidence on its health impacts is as yet scarce. Methods: we obtained, cleaned, and validated Particulate Matter (PM) concentration data from five air quality monitoring stations in the Skopje metropolitan area, applied relevant concentration-response functions, and evaluated health impacts against two theoretical policy scenarios. We then calculated the burden of disease attributable to PM and calculated the societal cost due to attributable mortality. Results: In 2012, long-term exposure to PM_2.5_ (49.2 μg/m^3^) caused an estimated 1199 premature deaths (CI95% 821–1519). The social cost of the predicted premature mortality in 2012 due to air pollution was estimated at between 570 and 1470 million euros. Moreover, PM_2.5_ was also estimated to be responsible for 547 hospital admissions (CI95% 104–977) from cardiovascular diseases, and 937 admissions (CI95% 937–1869) for respiratory disease that year. Reducing PM_2.5_ levels to the EU limit (25 μg/m^3^) could have averted an estimated 45% of PM-attributable mortality, while achieving the WHO Air Quality Guidelines (10 μg/m^3^) could have averted an estimated 77% of PM-attributable mortality. Both scenarios would also attain significant reductions in attributable respiratory and cardiovascular hospital admissions. Conclusions: Besides its health impacts in terms of increased premature mortality and hospitalizations, air pollution entails significant economic costs to the population of Skopje. Reductions in PM_2.5_ concentrations could provide substantial health and economic gains to the city.

## 1. Introduction

Air pollution causes significant health burdens worldwide [1]. Globally, the World Health Organization (WHO) estimates that 3 million premature deaths annually were attributable to ambient air pollution in 2012 [2]. About 480,000 of those premature deaths are estimated to have occurred in the WHO European Region, comprising 53 countries. The health effects of inhalable particulate matter (PM) are well documented. There is no evidence of a safe level of PM concentration [3], and more than 80% of the population in the WHO European Region lives in cities with levels of PM exceeding WHO Air Quality Guidelines [4]. In 2005, pollution from PM was estimated to reduce the typical life expectancy of an individual in the region by almost 9 months [4]. 

The former Yugoslav Republic of Macedonia (hereon MKD), and particularly Skopje—the capital and largest city of the country—have long suffered from poor air quality. In the WHO European region, MKD (37 μg/m^3^) ranks third after Bosnia and Herzegovina (42 μg/m^3^) and Tajikistan (41 μg/m^3^) for the annual population-weighted modelled urban and rural median concentration of PM_2.5_ [2]. In the 20th century Skopje has experienced rapid urbanization, growth of industrial activities, and reconstruction after the 1963 earthquake. With a comparatively large number of industrial point sources and higher traffic flows than other cities in the country, the Skopje agglomeration has historically experienced frequent episodes of heavy pollution [5,6]. During dry periods, the combination of mineral dust and emissions from residential heating, the transport sector, and industrial activities within the city increase the concentrations of inhalable particles [7,8]. 

Topography and meteorological conditions contribute to poor air quality in Skopje [9]. The city is situated in a valley and the river Vardar crosses the city, causing high humidity, particularly during the winter [10]. Additionally, heating facilities increase air pollution in winter [11]. An atmospheric anticyclone contributes to atmospheric temperature inversion, worsening ambient pollution levels, including particles with an aerodynamic diameter of less than 10 micrometres (PM_10_) and increasing hospital admissions among patients with cardiovascular diseases. An increase of 10 μg/m^3^ of PM_10_ above the currently maximum permitted values in the country (50 μg/m^3^ average over 24 h) increased daily admissions of patients with cardiovascular diseases during 2010 in Skopje by an estimated 12% [6]. In addition to the human health burden incurred, the morbidity and premature mortality due to air pollution entail significant economic and social costs. These include, but are not limited to, the cost to society of premature deaths, the costs of healthcare for the sick due to poor air quality, and the loss of productivity associated to that sickness and/or caregiving for oneself or others [12]. Thus, significant cost savings can be added to the health gains attainable through air pollution abatement. In this paper, we estimate for the city of Skopje the current health burdens and mitigation benefits of pollution on the local population in terms of hospital admissions and premature mortality, and in terms of their associated costs. The WHO air quality guidelines [13] and European Union (EU) reference values for PM_2.5_ (Directive 2008/50/EC) are used as counterfactual policy scenarios for a reduction of air pollution. 

## 2. Materials and Methods 

### 2.1. Selection of Pollutants and Data Processing

PM_2.5_ (or PM_10_) is an indicator for estimating health impacts of ambient air pollution mixture. PM is highly correlated with other air pollutants, such as NO_2_ from transport. PM exposure has been linked to a variety of adverse health outcomes, including short-term symptoms, chronic morbidity effects, and premature mortality [14]. For data validation and processing, we used the methods developed by the World Health Organization and others for health impact assessment of air pollution [15,16]. These methods identified criteria to select the appropriate monitoring stations. For particulate matter, a monitoring station was considered eligible for the study only if daily data were available for more than 50% of the study period; the daily average value of concentration was considered valid only if more than 50% of hourly data were available. 

In Skopje, five air quality monitoring stations were in operation at some point between 2005 and 2013, and these stations recorded PM_10_. Monitoring of PM_2.5_ was initiated in two of the stations in autumn 2011. Three stations have continuously monitored PM: Gazi Baba (urban background), Lisice (suburban industrial), and Rektorat (urban traffic) [17]. Overall, there was a high and significant correlation of measured PM_10_ for the 5 monitoring stations over the period 2012–2013. Average values measured by the monitoring stations for PM_2.5_ and PM_10_ were high (see Table 1), but availability of data were limited. The observed ratio PM_2.5_/PM_10_ was 0.610 for Centar and 0.609 for Karpos, both urban traffic monitoring stations.

### 2.2. Health Impact Assessment

#### 2.2.1. Population Data

The geographical area for the health impact assessment comprises the municipalities of Skopje in 2012 (not including Sopishte, physically segregated from the Skopje agglomeration). The target population considered is all residents exposed to the measured pollutants. This study focuses on health impacts of PM on the population of Skopje in 2012 and includes persons living in the surrounding municipalities of Skopje with the exception of Sopishte. According to official municipality statistics [18], the total population (not counting Sopishte) was 531,524 people, of which 260,216 (49%) were men (157,973 over the age of 30 years) and 271,308 were women (173,501 older than 30 years). Population data by district (ten in total, again not counting Sopishte) and five-year age groups were obtained from the Macedonian statistical office [19]. Mortality data (all-cause, non-accidental, disaggregated by ICD10 chapters) were obtained for the analysis period (2011–2013) from the Macedonian State Statistical Office, and morbidity data were obtained for the same years from the Skopje Center of Public Health.

#### 2.2.2. Selection of Health Endpoints

PM_2.5_ relative risks (RR) were used to estimate premature mortality in adults older than 30 years old from long-term exposure and to quantify health morbidity (Table 2).

Epidemiological calculations were carried out with WHO software AirQ+ [21,22]. The years of life lost (YLL) due to ambient air pollution were calculated using the life table calculator developed by Joseph Spadaro (obtainable upon request, see corresponding authors’ details.) and recently integrated into AirQ+. YLL are calculated for each specific calendar year in relation to a given population that is projected until its extinction, in this case after 105 years.

We computed PM_2.5_ impacts by calculating a population attributable fraction PAF, the proportion of incidence of specific health endpoints that is related to PM_2.5_ exposure:

PAF = 1 − 1/RR and RR = RRo^(∆C/10)^ (1)

in which RRo are the relative risks of the health effects listed in Table 2 (e.g., for long-term mortality 1.062) and ∆C is the change in ambient air concentration relative to a counterfactual scenario in μg/m^3^. The PM_2.5_ impacts are calculated as the product of the disease-specific PAF by the baseline mortality BM in the case of premature deaths (that is, premature deaths = PAF × BM), or the disease incidence rate if assessing health morbidity. There is no safe PM_2.5_ threshold level below which no negative effects are expected. However, a target concentration is needed to determine the attributable impacts or potential benefits of reducing the air pollution by a specified amount.

#### 2.2.3. Pollution Mitigation Scenarios

The following mitigation scenarios have been considered:

- Current situation—Annual average concentration of PM_2.5_ at 49.2 μg/m^3^
- Scenario 1: EU AQS—Annual average concentration of PM_2.5_ reduced to 25 μg/m^3^
- Scenario 2: WHO AQG—Annual average concentration of PM_2.5_ reduced to 10 μg/m^3^

Since PM_2.5_ data were incomplete, the “Current situation” estimate was calculated assuming a 0.61 conversion factor from PM_10_ to PM_2.5_. Scenario 1 is based on the air quality standards of the European Union (Directive 2008/50/EC) [23]. These seem like a sensible benchmark, since as an accession country, MKD would eventually be required to comply with EU standards. WHO air quality guidelines [13] recommend an annual average PM_2.5_ concentration equal to 10 μg/m^3^, which we chose for Scenario 2. 

### 2.3. Economic Evaluation of Premature Mortality

One approach to value the burden of premature mortality is to multiply the predicted number of deaths by the “value of a statistical life” VSL. The VSL represents society’s collective willingness to pay (WTP) for a small reduction in the annual mortality risk of death. The value of VSL is contextual: in this case, it refers to the value of preventing an anonymous premature death from exposure to air pollution. Various studies have derived VSL values in the European context [24,25,26]. A meta-analysis of VSL studies by [27] recommends for EU-27 a VSL of 3.6 million dollars (2005 prices), with an indicative range of 1.8 to 5.4 million dollars.

Ideally, national or regional studies should be used to value economic losses from exposure to ambient air pollution. In the absence of such studies, however, (2) may be used to transfer unit health costs (cost per case of illness or death) from a previous study to the policy location based on the “benefit-transfer” approach proposed by [27]. The adjustment takes into account differences in income levels between two places, all other socioeconomic conditions being similar (*ceteris paribus*).

(2) $$VSL_{p}=VSL_{s}{\left(\frac{Y_{p}}{Y_{s}}\right)}^{\beta }$$

Here, *Y* is the Gross Domestic Product GDP per capita (at purchasing power parity PPP prices), with subscripts *p* and s indicating policy (Skopje) and study (EU-27) locations, respectively. The coefficient *β* is the income elasticity factor, the marginal change in cost for a marginal change in income. In this study, we assumed *β* equals one, although other values have also been suggested in the literature [27,28]. Application of (2) to the case of Skopje yields 1.23 million euros (This value would be applied to deaths in year 2012). For all cost adjustments in this work, we assumed $13,500 and $34,500, respectively, for Skopje and EU-27 GDP per capita (PPP prices), and a purchase power parity exchange of 0.87 euros (2013) to the US dollar (2005). All costs in this study are stated in constant 2013 prices. The VSL varies year-to-year based on income growth and choice of discount rate. In this analysis we assumed a real growth rate (excluding inflation) of 2% per annum, whereas future costs and benefits have been discounted at a rate of 3% per annum.

Another approach to valuing premature deaths is to calculate the economic value attributed to the number of years lost, or loss of life expectancy, in the exposed population. For this type of assessment, we use the “value of a life year” (VOLY). Empirical values of VOLY have been determined using WTP studies in which respondents to questionnaires assigned monetary values to small gains in life expectancy, typically on the order of a few months, from mortality risk reductions in the context of exposure to ambient pollution. Unlike the VSL literature, only few studies in selected European countries have been carried out to date on the VOLY estimation. Further, these studies lack statistical strength due to the small sample sizes that were used in the analysis. Another frequent criticism in using this metric to value mortality impacts has been the lack of information on how VOLY might vary with age. For small changes in life expectancy, (i.e., on the order of months or at most a few years, such as would be the case of chronic exposure to air pollution) the VOLY has been used, while the VSL is the preferred valuation metric for accidental or immediate (also referred to acute mortality) deaths [29]. As the current population of Skopje ages over time, the mortality damage cost in future years varies depending on YLL and the VOLY. For our analysis, we chose a VOLY of 49,356 euros (2013 prices) in year 2012, calculated using the same benefit-transfer methodology applied to the VSL, and assuming a reference VOLY value of 126,000 euros for EU-27 (adapted from Holland, 2014). 

## 3. Results

### 3.1. Mortality and Ylls Attributable to Air Pollution in 2012 under Different Scenarios

At current pollution levels (49 μg/m^3^), the PM attributable mortality is 1199 premature deaths (CI95% 821–1519) in 2012. A hypothetical implementation of the EU limits could have averted an estimated 545 premature deaths, which is equivalent to a 45% decrease compared to the current situation, while a hypothetical reduction of PM levels to the WHO AQG could have averted an estimated 926 premature deaths (77% decrease). 

Regarding the years of life lost (YLL) by the 1199 estimated premature attributable deaths in 2012, these were estimated at 16,209 YLLs. This number is interpreted as the sum of lost years of life accrued by these individuals as a result of dying before their expected remaining lifetime at the time of death. The YLL per death equals 13.5, and the average loss of life expectancy among adults older than 30 years is 18 days (See Figure S1)—this figure applies to the population alive in year 2012 in this cohort and decreases as the population ages.

Overall, a significant number of YLLs could be prevented by policies that aim to mitigate air pollution. During the first year alone, the avoidable YLLs under Scenario 1 are 306, while the figure increases to 476 for Scenario 2. The life expectancy of the population at the current pollution level is 76.4 years at birth and 16.3 years for individuals older than 65 (See Table 3). 

**Figure 1:**
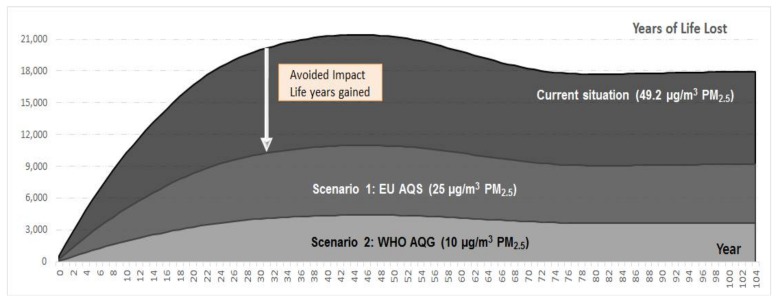
Health burden in terms of YLL and potential benefits under different mitigation scenarios.

The data listed in the table in columns “Number of deaths” and “Years of life lost” represent the current burden in year 2012 (“Current situation”), as well as the residual burdens under the two specified scenarios. In other words, under Scenario 2 (WHO AQG), there will still be 32.4 thousand deaths and 372 thousand YLL across the population aggregated over a time horizon of 105 years (i.e., the follow-up time until everyone alive in 2012 has died). Life expectancy increases as pollution levels are reduced (top to bottom in column 2). Thus, a shift from the current situation to Scenario 2 increases life expectancy at birth by 2.2 years. Compared to a hypothetical population unexposed to ambient pollution, even at the recommended WHO target there would still be a loss of life expectancy at birth of about one-half year (column 3). The long-term impacts of air pollution can be considered if the data are aggregated for a population alive in 2012 over the mentioned follow up period of 105 years, assuming a constant birth rate (See Figure 1).

### 3.2. Morbidity Attributable to Air Pollution in Year 2012 and Avoidable Impacts under Different Mitigation Scenarios

Morbidity outcomes were calculated for hospital admissions due to cardiovascular and respiratory diseases. The results under different scenarios are summarized in Table 4. Different proportions of the attributable morbidity in 2012 could have been “avoided” in scenarios with lower concentrations of PM_2.5_. Specifically, 19.9% of hospital admissions for cardiac disease and 19.6% for respiratory disease could have been “avoided” by achieving the EU standards; and 50.2% of hospital admissions for cardiac disease and 49.6% for respiratory disease could have been “avoided” by attaining the WHO air quality guidelines. 

### 3.3. Economic Benefits of Reduced Premature Mortality

**Figure 2:**
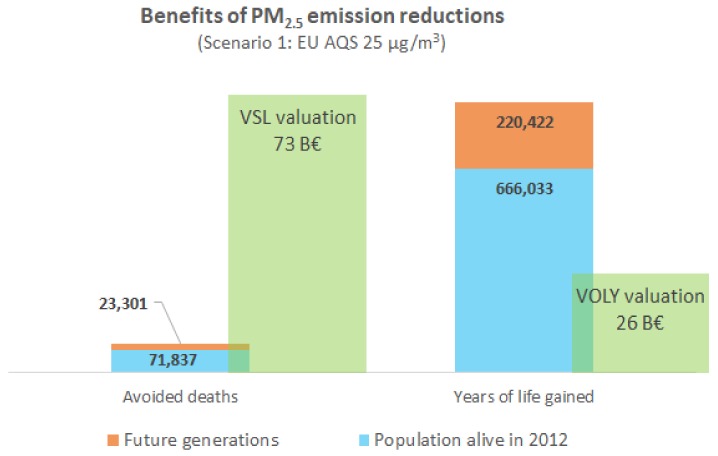
Scenario 1: cumulative mortality benefit of PM_2.5_ emission reductions for the city of Skopje aggregated over a follow up period of 105 years.

**Figure 3:**
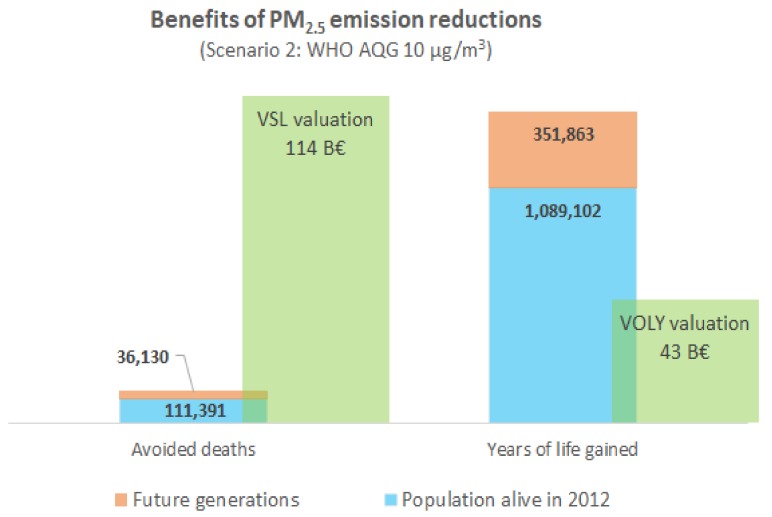
Scenario 2: Cumulative mortality benefit of PM_2.5_ emission reductions for the city of Skopje aggregated over a follow up period of 105 years.

The social cost for the estimated premature mortality attributable to PM_2.5_ in the year 2012 in Skopje was between 570 M€ (VOLY metric) and 1470 M€ (VSL metric). Significant cost savings at the social level could be attained through air pollution abatement. Assuming a long-term real income growth of 2% and a 3% discount rate, the mean annual benefit of the mortality reduction achieved through Scenario 1 is between 251 M€ (using the VOLY metric) and 697 M€ (VSL metric). For scenario 2, the annual accrued benefit would be between 407 M€ (using the VOLY metric) and 1081 M€ (VSL metric). The health benefits and social costs of the two alternative scenarios can be visualized in a distribution across the population by distinguishing the present generation (population alive in 2012) versus future generations (up to 105 years into the future). Over a follow-up period of 105 years of the population alive in Skopje in the year 2012, and also accounting for future generations, the accumulated benefits of avoided premature mortality due to air pollution are substantial (see Figure 2 and Figure 3). With a VOLY valuation, the cumulative benefits would be of 26.3 billion € (2013 prices) under Scenario 1 and 42.7 billion € under Scenario 2. With VSL valuation, the benefits would be 73 billion € (2013 prices) under Scenario 1 and 114 billion € under Scenario 2. 

## 4. Discussion

In the year 2012, at current pollution levels (49 μg/m^3^), we estimated that PM_2.5_ may have caused an estimated attributable mortality of 1199 deaths, as well as 547 hospital admissions for cardiovascular diseases and 937 hospital admissions for respiratory diseases, with a social cost of between 570 M€ and 1470 M€. Of all these estimated impacts, a hypothetical implementation of the EU limits could have averted an estimated 45% of premature deaths, about 20% of hospital admissions both for cardiovascular and respiratory diseases, and yielded social cost savings of between 251 M€ and 697 M€. A hypothetical further reduction of PM levels to the WHO AQG could have averted an estimated 77% of premature deaths, about 50% of hospital admissions for both cardiac disease and respiratory disease, and yielded social cost savings of between 407 M€ and 1081 M€. Consistently, the accumulated benefits of avoided premature mortality over the long term due to air pollution would be substantial.

Both the health impact assessment and the health economic valuation of air pollution in Skopje are generally higher than previous results for MKD as a whole. The World Bank [30] estimated that approximately 1350 premature deaths were attributable to inhalable particulate matter in MKD in 2011, with an economic cost of €253 million annually or 3.2% of the national GDP. Our results for Skopje in terms of attributable mortality would thus represent almost 90% of the World Bank estimate for the whole country, of which only about a quarter of the population lives in the capital. Although that study did not show the downscaled mortality burden to the urban level, it did show the disaggregated health-related economic burden, including VSL-based mortality cost, of air pollution to be heavily concentrated in Skopje (45% of the total). A related paper by the same team [31] elaborated further on the methodology of the World Bank study. Their RR functions, taken from an older review [16] are for cause-specific endpoints (long-term cardiopulmonary and lung mortality) compared to the all-cause natural mortality RR that we used [14]. Further, we assume no counterfactual for the current situation, but there is a recommended cutoff (7.5 μg/m^3^ for PM_2.5_) in the mentioned review. These factors would suggest a smaller difference between the World Bank estimates and ours than expected on the basis of population and measured pollution concentrations alone.

Another study [32] provides an estimate in 2013 of 3360 deaths attributable to PM_2.5_ (annual mean 30.4 μg/m^3^ ) for the whole country. Adjusting for the difference in share of the population 30+ in Skopje and nationally (62.4% and 59% respectively), as well as for the differences in exposure (urban in Skopje vs. a mix of urban and rural nationally), their adjusted premature mortality estimate is within 15% of our result. 

Our study has a number of limitations. We have assumed a log-linear function between exposure and response to ambient pollution, so health effects would be the same for incremental ranges of pollution at different levels. For the relatively high exposures experienced in Skopje, this is likely to result in an over-estimation of the burden, as well as of the health co-benefits associated with reduced pollution. On the other hand, however, our results should be interpreted as conservative. For instance, some relevant pollutants were excluded from the analysis (e.g., ozone and NO_2_), although their relative contribution to health morbidity and mortality is likely to be relatively small compared with PM [14]. Furthermore, some important nonfatal health endpoints such as aggravation of asthma and other chronic respiratory diseases, which may in turn impact a person’s ability to work or engage in routine daily activities, were excluded, but could represent significant additional health and economic burdens on the population and social services. In past European studies, morbidity costs accounted for 10 to 15% of the total health cost when mortality was valued using VSL [12].

The data availability for our study was not optimal, with a very low coverage of measurements for most stations in 2011, and to a lesser extent in 2013. These periods of non-functionality of the measuring stations were officially noted at the yearly reports of the Macedonian Ministry of the Environment, hinting at the urgent need for the air quality monitoring system in Skopje to be strengthened, particularly the geographical and time series coverage of measurements, as well as the availability of adequate human and physical resources for monitoring.

Notwithstanding these limitations, both our results and previous ones highlight the urgency of reducing the exposure to particulate matter in urban settings in MKD. The World Bank [30] provided policy advice for such reduction tailored to the emissions profile of the country, targeting pollution abatement in the industrial sector, a quick transition from the current lignite-based energy generation to a gas-based one, a mix of sustainable transportation policies, efficient residential stoves and boilers, and an adequate system of incentives for compliance at all levels. These are aligned with the Macedonian Government EU-backed regulatory efforts towards compliance with the EU environmental acquis, making the issue of implementation the most pressing concern. Such implementation is likely to be more sustainable and effective if it is supported by adequate multi-level governance structures and inclusive policy processes [33]. 

## 5. Conclusions

Inhalable particulate matter causes significant mortality and illness in Skopje. The average life expectancy of the residents of Skopje is being reduced by 2 to 3 years through a largely preventable environmental factor like anthropogenic air pollution. It also entails a sizeable welfare cost to society. While there is no safe level of PM, any decrease in concentrations will benefit health in Skopje by decreasing premature mortality and morbidity, and related economic costs. The attainment of the European Union standards for PM can provide substantial health and economic benefits, but the attainment of WHO air quality guidelines levels can provide further benefits and should eventually constitute a policy goal. Our results underscore the urgency of implementation of measures to reduce emissions and, ultimately, exposure.

Even a limited assessment like the one featured in this work can provide useful evidence for policymaking. This is currently very relevant, as MKD continues to align its regulations with the EU acquis [34] on environmental issues and is in the process of implementing a National Environmental Health Action Plan to tackle the main environmental concerns in the country. More research and local evaluations on the health and economic impacts of urban air pollution are needed, particularly in EU accession countries and other neighboring countries in which the local evidence base on this topic is scarce. 

## Figures and Tables

**Table 1:** Data availability and mean values by pollutant and monitoring station in Skopje for three consecutive years.

	Centar (Traffic)			Gazi Baba (Background)			Karpos (Traffic)			Lisice (Industrial)			Rektorat (Traffic)		
**PM_2.5_ (mean, µg/m^3^)**	**48.3**			n.a.			**41.6**			n.a.			n.a.		
13 December 2011 availability (% per year)	28	91	85	n.a.			29	97	50	n.a.			n.a.		
**PM_10_** **(mean, µg/m^3^)**	**79.2**			**84.4**			**68.3**			**108.6**			**71.7**		
13 December 2011 availability (% per year)	28	91	90	3	58	58	29	97	91	86	92	98	21	96	99

**Table 2:** Selected air quality indicators and PM relative risks.

Health Endpoint (Specific Population)	ICD10 Codes	Relative Risk (RR)	Source
All-cause mortality excl. accidents (Adults 30 years and older)	A00-R99	For 10 μg/m^3^ increase in PM_2.5_ RR = 1.062 (95% CI: 1.040–1.083)	[20]
Hospital admission for cardiovascular diseases (all ages)	I00-I52	For 10 µg/m^3^ increases in PM_2.5_ RR = 1.0091 (95% CI: 1.0017–1.0166)	Air Pollution Epidemiology Database—APED [14]
Hospital admissions for respiratory diseases (all ages)	J00-J99	For 10 µg/m^3^ increases in PM_2.5_ RR = 1.0190 (95% CI: 1.0190–1.0402)	Air Pollution Epidemiology Database—APED [14]

ICD10: International Classification of Diseases 10th revision.

**Table 3:** Health burdens of the different PM_2.5_ scenarios, expressed as aggregate impact over a follow-up period of 105 years.

PM2.5 Ambient Concentrations	Life Expectancy (Years)	Loss of Life Expectancy * (Years)	Number of Deaths (Thousands) **	Years of Life Lost (Thousands)
Current situation: 49.2 μg/m^3^	76.4 (at birth)	2.8 (at birth)	179.9	1813
	16.3 (65 years)	2.1 (65 years)	(123.2−227.9)	(1177−2413)
Scenario 1: EU AQS (25 μg/m^3^)	77.7 (at birth)	1.4 (at birth)	84.8	926.5
	17.3 (65 years)	1.1 (65 years)	(59.7−104.6)	(600.3−1235)
Scenario 2: WHO AQG (10 μg/m^3^)	78.6 (at birth)	0.6 (at birth)	32.4	372.0
	18.0 (65 years)	0.5 (65 years)	(23.2−39.4)	(240.7−496.6)

* Loss of life expectancy compared to a hypothetical unexposed population (life expectancy = 79.15 years at birth and 18.45 years at age 65) ** Values in brackets show 95% confidence intervals of estimates based solely on the RR uncertainty interval indicated in Table 2.

**Table 4:** Morbidity outcomes under different PM_2.5_ scenarios in year 2012.

Morbidity	Current Situation	Scenario 1 EU Limits (95% CI)	Scenario 2 WHO AQG (95% CI)
Hospital admission for cardiovascular diseases	547 (104–977)	438 (83–784)	272 (51–490)
Hospital admissions for respiratory disease	937 (937–1869)	753 (753–1516)	472 (472–964)

## Supplementary Materials

The following are available online at http://www.mdpi.com/1660-4601/15/4/626/s1. Figure S1—Time trend of mid-year population stratified by age under current conditions; Figure S2—Costs of PM_2.5_ concentrations and mitigation scenarios for the city of Skopje; Figure S3—Cumulative mortality cost based on YLL for current pollution level.
