# Supplementary material for: Health Impacts and Economic Costs of Air Pollution in the Metropolitan Area of Skopje
Source: Int J Environ Res Public Health. 2018 Mar 29;15(4):626. doi: 10.3390/ijerph15040626 (PMC5923668; doi:10.3390/ijerph15040626)

Supplementary material

Authors: Gerardo Sanchez Martinez, Joseph V. Spadaro, Dimitrios Chapizanis, Vladimir Kendrovski, Mihail Kochubovski and Pierpaolo Mudu

Manuscript title: Health impacts and economic costs of air pollution in the metropolitan area of Skopje

Pages: 2

Figures: 3

Figure S1 – Time trend of mid-year population stratified by age under current conditions

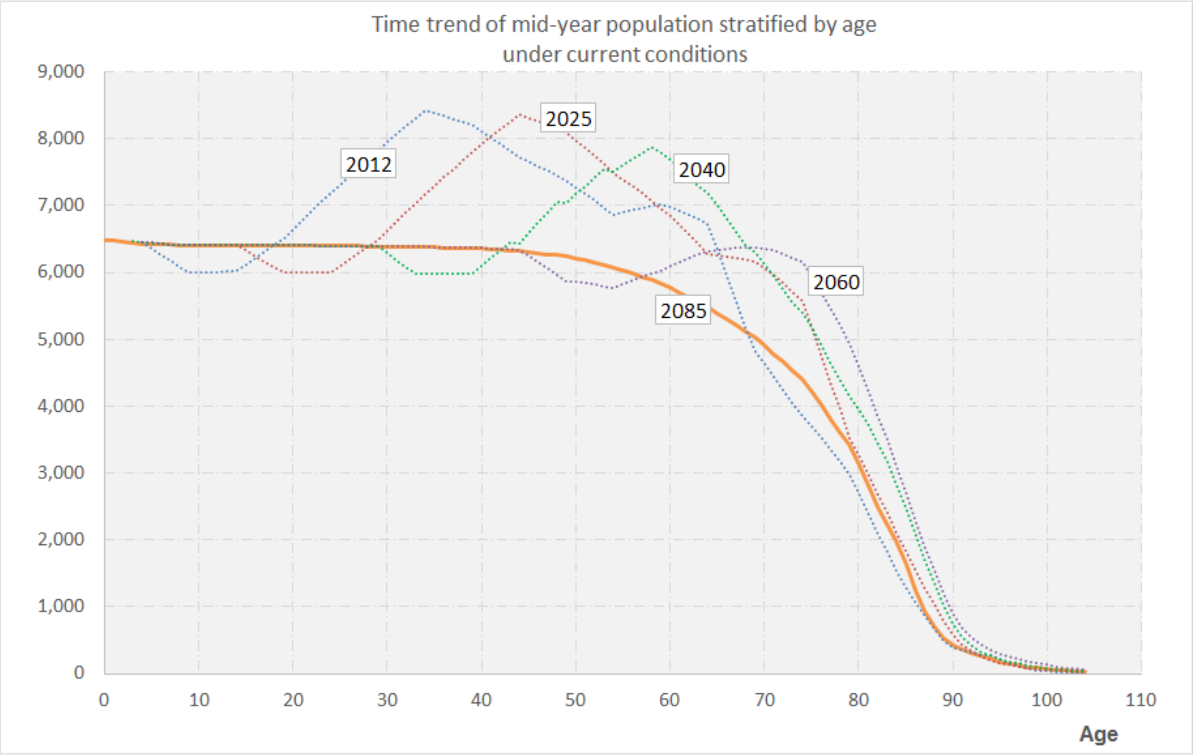

Figure S2 - Costs of $PM_{2.5}$ concentrations and mitigation scenarios for the city of Skopje

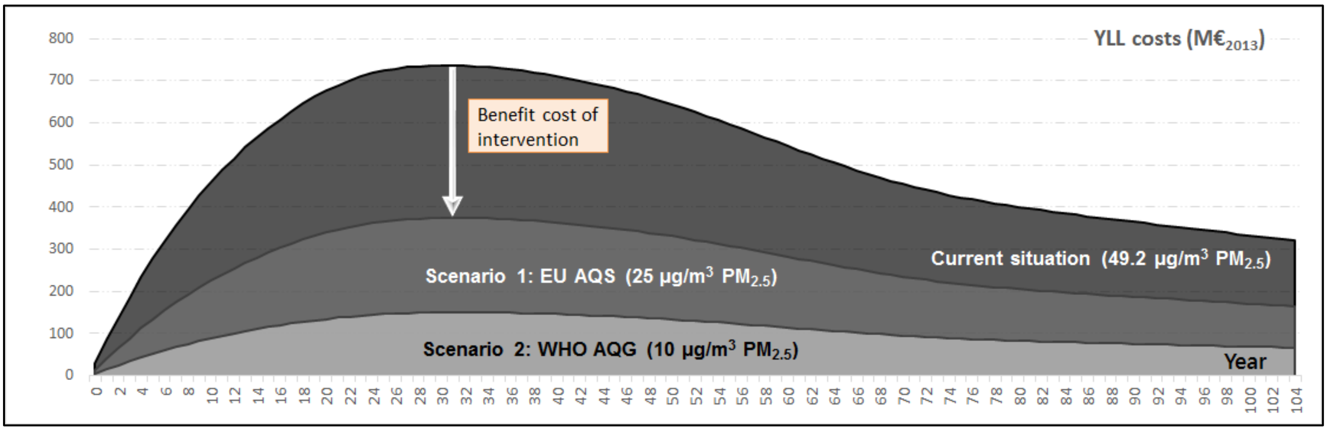

Figure S3 – Cumulative mortality cost based on YLL for current pollution level

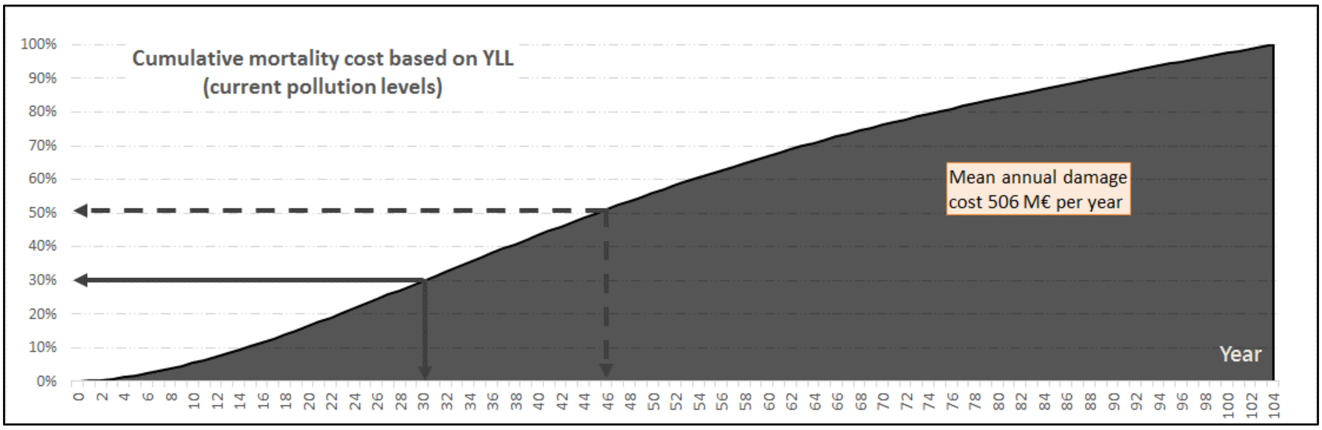

Supplement: Supplementary file 1 [file ijerph-15-00626-s001.pdf]
